# Family experiences in managing mental disorders in adolescents

**DOI:** 10.1590/0034-7167-2025-0312

**Published:** 2026-07-24

**Authors:** Roberto Corrêa Leite, Maria Giovana Borges Saidel, Edirlei Machado dos Santos, Heloisa Garcia Claro Fernandes, Luciana de Lione Melo, Maria Cristina Mazzaia, Michelle Ferraz Martins Jamarim, Claudinei José Gomes Campos

**Affiliations:** I Universidade Estadual de Campinas. Campinas, São Paulo, Brazil; II Universidade Federal de Mato Grosso do Sul. Três Lagoas, Mato Grosso do Sul, Brazil; III Universidade Federal de São Paulo. São Paulo, São Paulo, Brazil

**Keywords:** Adolescent, Mental Disorders, Family, Family Relations, Mental Health., Adolescente, Trastornos Mentales, Familia, Relaciones Familiares, Salud Mental.

## Abstract

**Objectives:**

to understand the family management experience of mental disorders in adolescents in light of the Family Management Style Framework.

**Methods:**

a qualitative case study was conducted with six families between July and November 2022. The interviews were analyzed using a hybrid thematic analysis model, with the support of NVivo^®^ 15.

**Results:**

families understand mental illness as a condition marked by stigma and daily challenges. Adolescents’ identity oscillates between normality and vulnerability, guiding care towards safety and well-being. Changes in routine reflect the effort to cope with unpredictability, while future expectations value adolescents’ autonomy and mental health.

**Final Considerations:**

the study provides support for interventions that promote resilience and family functionality, based on psychoeducation, problem-solving, reinforcement of strengths, and mitigation of problematic aspects of family management.

## RESUMEN

**Objetivos:**

comprender la experiencia familiar en la gestión de trastornos mentales en adolescentes a la luz del Family Management Style Framework.

**Métodos:**

estudio de caso cualitativo con seis familias entre julio y noviembre de 2022. Las entrevistas se analizaron mediante un modelo híbrido de análisis temático, con el apoyo de NVivo^®^ 15.

**Resultados:**

las familias comprenden la enfermedad mental como una condición marcada por el estigma y los desafíos cotidianos. La identidad del adolescente oscila entre la normalidad y vulnerabilidad, orientando la atención hacia la seguridad y el bienestar. Los cambios en la rutina reflejan el esfuerzo por afrontar la imprevisibilidad, mientras que las expectativas futuras valoran la autonomía y la salud mental del adolescente.

**Consideraciones Finales:**

el estudio respalda intervenciones que promueven la resiliencia y funcionalidad familiar, basadas en la psicoeducación, la resolución de problemas, el fortalecimiento de las fortalezas y la mitigación de los aspectos problemáticos de la gestión familiar.

## INTRODUCTION

Biopsychosocial changes during adolescence can compromise brain development, favoring the emergence of mental disorders^(1)^. In this scenario, emotional regulation is frequently affected, making it difficult to tolerate intense and persistent emotional states^(2)^. Emotional dysregulation is associated with suicidal ideation and attempts^(3)^, as well as behaviors such as mood instability, anger outbursts and aggression^(2)^, representing a challenge for adolescents and their families.

In 2021, about one in six young Brazilians between the ages of 10 and 19 lived with some kind of mental disorder^(4)^, making this the age group most vulnerable to self-harm, depression, and suicide. Half of the cases appear around the age of 14, but remain undiagnosed or without professional follow-up. Depression is among the leading causes of disability, and suicide is the third leading cause of death among young people aged 15 to 29^(1)^.

Given these conditions, family management style influences individual and family system adaptation^(5)^. In the case of mental disorders, parents face severe symptoms such as hallucinations, delusions, isolation, and mood swings^(6)^. More effective strategies include emotional support, appropriate treatment and empathetic interactions, while control attitudes aimed at changing behavior prove less effective^(7)^. Family management refers to the family’s actions and responses in the face of illness and healthcare, being central to the Family Management Style Framework (FMSF)^(8)^, which comprises three components: definition of the situation; management behaviors; and perceived consequences. This model makes it possible to understand how families cope with chronic conditions throughout the life cycle^(5)^.

Defining the situation encompasses subjective meanings about care, including adolescents’ identity, perspective on the condition, management mindset, and parental mutuality. Management behaviors relate to everyday conduct, such as parenting philosophy and caregiving approach. Perceived consequences refer to current impacts and future expectations of the family^(5)^.

Although the FMSF is widely applied in chronic conditions^(8)^, its use in the context of mental disorders in adolescence is still incipient. This study adopts the FMSF to understand how families manage the daily care of adolescents with mental disorders, highlighting the need for healthcare professionals to be prepared to support the management of these complex situations.

## OBJECTIVES

To understand the family experience of managing mental disorders in adolescents in light of the FMSF.

## METHODS

### Ethical aspects

The study was approved by the *Universidade Estadual de Campinas* (UNICAMP) Research Ethics Committee, according to the Certificate of Presentation for Ethical Consideration 47887321.6.0000.5404. The interviews were conducted only after the reading and signing of an Informed Consent Form (ICF) by participating families.

### Study design

This is qualitative research^(9)^, which used the FMSF^(8)^ as a theoretical framework, conducted through a multiple and holistic case study, appropriate for the in-depth investigation of phenomena in real-life contexts^(10)^. Families constituted the context of the cases, with family management as the unit of analysis. The study followed the guidelines of the Consolidated Criteria for Reporting Qualitative Research (COREQ), recommended for design and analysis in qualitative research.

### Study setting

The study was conducted in a virtual environment, within the national territory and the home of the participating family. A location was chosen where both participant and researcher felt safest, most comfortable, and with guaranteed privacy.

### Data source

Families with at least one adolescent between 13 and 17 years old, with a medical diagnosis of mental disorder confirmed by a psychiatrist, under outpatient or therapeutic follow-up for at least six months, were included. Families had to reside within the national territory, have internet access, and be able to participate in virtual interviews. Families whose adolescents presented severe intellectual disabilities that made communication impossible, or those who, after the first contact, expressed discomfort or withdrew from participating in the study, were excluded. Data collection was carried out via the Zoom^®^ platform, in accordance with guidelines for virtual research^(11)^, through two semi-structured interviews with each family, ensuring data credibility, reliability and triangulation^(10)^. The script followed the FMSF dimensions: definition of the situation; management behaviors; and perceived consequences^(5)^. Six families with adolescent children diagnosed with mental disorders participated in the study. These families resided in different regions of Brazil and had internet access. Adolescents were identified by fictitious names (Adam, Felipe, Sofia, Henrique, Aurora, and Bela), and parents by their initials and relationship (e.g., “Mother” or “Father”).

### Data collection and organization

Data collection was carried out by the principal researcher between July and November 2022 in three stages. The first stage included an interview for the elaboration of genograms, ecomaps^(12)^, and life history of families. In the second stage, semi-structured interviews based on the FMSF were applied, with an average duration of 78 minutes. The third stage consisted of telephone contact for clarifications and case closure. Two interviews were conducted via Zoom^®^ with each of the six families, with 11 individual interviews and one interview with a couple, totaling 12 interviews with seven family members (six mothers and one father). The audio recordings were transcribed and validated via WhatsApp^®^, without corrections. The sample was intentional, with snowball recruitment^(13)^ and closed when the interviews reached theoretical saturation^(9)^. The first family (Adam’s Family) was referred by a mental health nurse specialist and, like the others, received an invitation to participate in the research via a letter sent through WhatsApp^®^, after confirmation of inclusion criteria. The invitation contained preliminary information about the research, as well as a Google Forms^®^ link to access the ICF. After consent to the ICF, evidenced by participants’ acceptance and digital signature, the date for the first interview was scheduled. To confirm theoretical saturation, two additional interviews (one individual and one with a couple) were conducted after the fourth family. As no new topics or relevant contributions to the analytical categories emerged from these interviews, theoretical saturation was confirmed, evidenced by repetition of narratives and stability of interpretations.

### Data analysis

A hybrid model of thematic analysis was chosen, which combines deductive and inductive coding to identify topics relevant to the phenomenon under study^(14)^. Deductive codes were developed based on the FMSF^(5)^ dimensions and organized into a template in NVivo^®^ using a structural matrix. Inductive analysis allowed the inclusion of the “mutuality in the family” code, supported by the FMSF^(5,8)^ authors and by studies with families in other chronic conditions^(15-17)^. Transcripts were exhaustively coded, and the topics were analyzed and interpreted in light of the theoretical framework. Word clouds in NVivo^®^ were also used as a complementary interpretation tool. The analysis was conducted by the principal investigator and validated by members of the Research and Qualitative Studies in Health Center at UNICAMP.

## RESULTS

**Figure 1:**
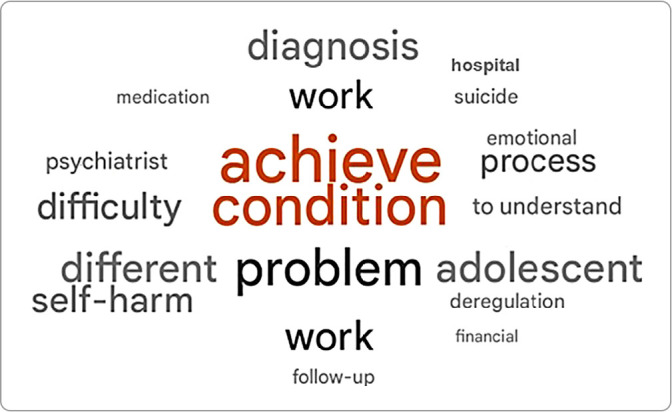
Cloud of the 20 most frequently cited words, with a minimum length of eight characters, in the interviews with families participating in the research, Campinas, São Paulo, Brazil, 2025

Seven family members of six adolescents with mental disorders were interviewed. These individuals resided in São Paulo (three families), Paraíba, Rio de Janeiro, and Pernambuco. Participants were between 36 and 45 years old, comprising six mothers and one father, mostly from nuclear families (three), in addition to two remarried families and one adoptive family. All had completed higher education and had access to health insurance. Adolescents, aged between 13 and 17, included a transgender man, two boys, and three girls, diagnosed with autism spectrum disorder, attention deficit hyperactivity disorder, bipolar affective disorder, generalized anxiety disorder, depression, and borderline personality disorder. In all families, mothers were the adolescents’ primary caregivers, but in Felipe and Bela’s families, grandparents also provided support. NVivo^®^ analysis highlighted the “condition” word as the most recurrent (0.23%) in the word cloud (Figure 1), reflecting its centrality in the family experience and its articulation with the other meanings evoked in the *verbatims*.

The family management experience was analyzed based on the FMSF components and dimensions. Concerning adolescents’ identity, some families adopted a more optimistic view, associating the condition with possible normality. Others emphasized limitations and impacts on socialization, psychosocial aspects, and dysfunctional behaviors.

[...] *so, aside from the specific needs related to his* [Felipe’s] *disorders, which are certain, I try to see him in a normal way and recognize his desires for socialization and the difficulties he has, just like other adolescents.* (Mother, Felipe’s Family)

*So, I think we* [parents] *define Aurora’s adolescence as having been stolen. Totally stolen, because it was gone* [...] *she wasn’t going out much anymore, and her friends were starting that phase of 15^th^ birthday parties* [...]. *Then she got sick; her friends* [adolescent friends] *continued with their lives; she didn’t!* [...] *but that’s how it is, she missed that phase.* (Mother, Aurora’s Family)

*I think he* [Henrique] *doesn’t experience many adolescent things. His mind works in a very different way.* [...] *once he told me that he has to try hard not to think about killing himself; that, for me, is already a very sad thing to hear from an adolescent. So, I think his worries are too big for a 16-year-old boy.* (Mother, Henrique’s Family)

Families’ perspectives on the condition are impactful and permeated by intense feelings related to the cause, severity, and prognosis of the mental disorder. Their journey involves grieving for the idealized child, guilt, stigma, and the search for knowledge to better understand and manage the situation.

*I found out about the diagnosis when I was 2 years old. Horrible, horrible, horrible! I was very upset* [...] *I went to work on autopilot, I suffered a lot* [...] *so, emotionally, I think I went through a grieving process.* (Mother, Adam’s Family)

*Sometimes, I’m like that, Roberto* [researcher]. *I keep saying, “Where did I go wrong? What did I do wrong? Where was it? At what point?”. Trying to identify it chronologically* [...]. (Father, Bela’s Family)

*It* [mental disorder] *is not seen. It is not talked about, I won’t generalize, but for a large number of people, it’s a problem that seems trivial. Let it go, get up, dust yourself off and let’s go* [...] *that’s it. It’s in the workplace of someone who has burnout or depression, or at school, as she* [Aurora] *experienced*. (Mother, Aurora’s Family)

[...] *I started researching the subject and began to understand the illness and the needs a little better. So, psychoeducation is very necessary,* [...] *there is no psychoeducation for parents.* [...] *in fact, it was a very important point for me when I joined Family Connections* [skills training for caregivers of people with mental health conditions] *as a family member. I thought, “Wow, so many people are going through so many similar things to us, aren’t they?”. They have the same doubts we all have* [...]. (Mother, Felipe’s Family)

A parenting mindset reveals how parents perceive their abilities, challenges, and support in childcare. Despite exhaustion and uncertainty, they recognize parenting as an ongoing process that requires patience and perseverance to ensure their children’s safety.

*It’s very difficult, really very difficult* [taking care of Sofia]! *I thought I wouldn’t have the strength, the discernment to do the things I have to do today. It’s a kind of care where you have to think about everything, everything you’re going to do, from a simple bath, where she could cut herself, to putting her to sleep, where she could wake up, and so it’s very, very difficult! Everything we propose is met with a “no”! So, we have to go very slowly, sometimes not go, sometimes not do, and that’s how we live.* (Mother, Sofia’s Family)

The difficulty in controlling emotional dysregulation crises and predicting their triggers generates fear, frustration, and anxiety, keeping parents in a state of hypervigilance. Families also report a lack of support from public authorities and healthcare professionals in the face of psychological suffering and the psychosocial impact of care.

*And I begged her* [Bela], *for the love of God, sobbing, not to do it anymore* [self-harm], *not to try* […] *just talking about it makes my eyes water! That’s it, sometimes it’s hard to identify the trigger, what can or cannot cause that, let’s say, that’s the trigger, isn’t it?* (Father, Bela’s Family)

*Extremely exhausting* [taking care of Felipe]. *It has an impact on your personal life, professional life, and your physical and mental health, a profound impact, and we do this with less and less help from the public authorities. Mental health is still seen as your problem, and there’s zero support here* [in the city where she lives], *from the ambulance service to the place you take him.* [...] *where are we, right? The people* [healthcare professionals], *who are supposed to be specialized in dealing with something like this, what a difficulty!* (Mother, Felipe’s Family)

According to parental mutuality, parents became more united in the experience, resulting in greater connection, communication, and reciprocity between the couple.

[...] *we’ve always been a very close couple, but I think now the pain* [of Aurora’s condition], *right? Pain makes you stay together and endure, and support each other.* (Mother, Aurora’s Family)

*More unity! I think the relationship was already a very solid relationship, and when you have a child, whatever condition they have, if it’s not a solid relationship, there isn’t that giving and that sharing* [...] *and I have a lot of support from my husband, thank God!* (Mother, Adam’s Family)

In other families, the management of the condition was centered on the extended family or stepfather, due to the absence of the biological father, which characterized familial mutualism.

*But today we* [the extended family] *are also united and quite strong in that aspect as well.* [...] *and today we are, I think, also because of everything we’ve been through, even more united.* (Mother, Felipe’s Family)

*It ends up being very much just the two of us* [she and her stepfather] [...] *we talk a lot about everything. I try to keep him always informed about what I find out, about what’s happening* [...]. (Mother, Henrique’s Family)

Parenting behaviors reflect parental philosophy and the approach adopted. All families demonstrated a strong belief in the effectiveness of multidisciplinary interventions and a commitment to the emotional, cognitive, and behavioral development of their children.

*But we took her to another specialist doctor, and that’s when he started working with the hypothesis of borderline personality disorder. He changed the medications, but even then, it didn’t work, and we had to admit her to the first clinic.* (Mother, Sofia’s Family)

[...] *is a speech therapist, an individual behavioral psychologist, and then I started digging for my rights, looking for what was best.* (Mother, Adam’s Family)

Mothers live in a constant state of readiness, always expecting the next crisis, and face unpredictability in their daily lives. These experiences result in emotional exhaustion and negatively impact their quality of life. Therefore, they focus on self-care strategies to strengthen their mental health, given the physical and emotional overload they face caring for adolescents.

*I tend to think that we’re always on alert, always expecting the next crisis, expecting the next situation, expecting what will happen, how it will be. Now I’m managing to deal with it a little better, with therapy, but, a while ago, I always had it in my head that at any moment someone would call me and say that my son had done something stupid* [suicide attempt]. (Mother, Henrique’s Family)

*Because I didn’t see any light at the end of the tunnel anymore. I was already in a state of despair! I even had many suicidal thoughts. Not the kind of thought that you act on, that you plan* [...] *but the kind of thing you wish for. I would have preferred to be dead for having gone through all this. You understand?* [...] *so, I sought help, because otherwise, I wouldn’t have been able to help him* [Felipe]. (Mother, Felipe’s Family)

The management approach showed that mothers adjusted their work routines to accompany adolescents to appointments and therapies, ensuring continuous support and preventing risky behaviors. Dysregulation crises also required changes in family dynamics, with an emphasis on observing boundaries and empathetic communication.

*I’ve already given up jobs. I’ve already chosen others, because F.* [Father], *since he has an administrative position, always works those hours, from 8:00 AM to 5:00 PM, but I’ve already changed jobs* [...] *I’ve had a six-hour job, from 2:00 PM to 8:00 PM, to provide support in the morning. Anyway, it was along those lines. I’ve already given up, I’ve already stayed home, I’ve already quit* [resigned]. (Mother, Adam’s Family)

[...] *so, I already had to be looking for doctors and psychologists. There were problems with him* [Felipe], *problems at school, and it demanded so much of my time that I couldn’t reconcile it with my professional life. When I tried* [to work], *I saw such a big worsening in the boy that I had to stop.* (Mother, Felipe’s Family)

*That gentle reprimand, and then you say, “Let’s not go in like a bull in a China shop, we have to go a little slower, so as not to be the trigger”* [emotional dysregulation]. *I think it’s about having that sensitivity to take it a little easier* […]. (Father, Bela’s Family)

Perceived consequences involve families focusing on the care of adolescents amidst unpredictability and daily disruptions. The routine demands cautious actions, often described as “walking on eggshells”. Even so, some mothers report progressive adjustments over time, with a positive perception of the changes.

*Ultimately, you can’t have a very stable and emotionally structured life for very long. It seems like something is always going to change and you have to keep adjusting your plans* [...]. (Mother, Adam’s Family)

[...] *everything, everything, everything, everything you can imagine has changed. I think nothing is the same as before* [...] *even though she* [Sofia] *is hospitalized, today we still continue with the same care routine* [...] *afraid that the phone will ring and we’ll have to rush out. It’s what we always hear. It seems like we’re living to walk on eggshells all the time.* (Mother, Sofia’s Family)

*It totally interferes with everything* [care for Felipe] [...] *so, it’s a completely different routine*. [...] *today, when I got home,* [...] *I saw that he had hung up his clothes, that he had taken his medicine, without me telling him anything, because I was sleeping. So, I’m very grateful for everything, because I know what we* [the family] *have already been through* [...]. (Mother, Felipe’s Family)

The analysis of future expectations reveals the desire for adolescents to achieve autonomy and independence, while also expressing parents’ fear and concern regarding the possibility of treatment interruption and the consequent increased risk of suicide.

*My expectation for Adam is that he will be functional! That he will be able to perform basic activities, to get around independently. I think that’s it! Regarding my family, it’s about following his developmental process, having the satisfaction of seeing in the future that nothing, absolutely nothing, was in vain. You understand? That everything turned out alright!* (Mother, Adam’s Family)

[...] *I want her* [Aurora] *to be independent, to not depend on anyone, to go and do things. She can come to my house, that’s fine, she can be here, but she has her own life, she has her own children. She can bring them for me to see. I don’t want to interfere either* [...]. (Mother, Aurora’s Family)

*But do you have any expectations regarding Sofia’s future?* (Researcher)

*No, I just want her to stay alive! I want her to stay alive! Because I’m sure that from the moment she stops taking the lithium* [medication], *she’ll go back to having suicidal thoughts.* (Mother, Sofia’s Family)

## DISCUSSION

Deductive and inductive analysis, guided by the FMSF, revealed that adolescents’ identity oscillates between perceptions of normality and limitations imposed by the mental disorder. Normalization emerged as a coping and adaptation strategy to daily life. Conversely, the constant fear of crises and life-threatening behaviors compromised family functionality, intensifying vigilance. These findings are consistent with the literature, which recognizes normalization as a coping resource^(18)^, while continuous exposure to suffering tends to weaken families’ adaptive resources^(19,20)^.

The view of the condition showed the diagnosis as a painful rupture associated with grief^(21)^ and the search for knowledge^(22)^. Denial appeared as an initial response to the loss of the idealized child, giving way, over time, to acceptance. These results converge with studies that understand denial as a defense mechanism and a transitional phase of parental grief^(12,21,23,24)^. These studies highlight professionals’ importance, especially nurses, recognizing this movement as part of the adaptive process, offering support with validating listening and sensitive presence.

Guilt was recurrent in the reports, reflecting parental responsibility linked to a lack of knowledge about the condition. Beliefs that children’s development depends solely on the love and effort of parents intensified this feeling, leading to internalization of suffering^(19,25,26)^. Nurses play a fundamental role in welcoming families with qualified listening and clear information to alleviate guilt and give new meaning to the parental experience.

Moreover, participants reported feeling helpless in the face of the invisibility and social delegitimization of mental health conditions. Such perceptions were associated with the experience of stigma and the feeling of not belonging, which can be aggravated by a biomedical model that tends to categorize individuals as different, contributing to the weakening of social bonds, family resilience, and the recovery process of adolescents^(21,27)^.

The lack of professional guidance led families to actively seek sources of knowledge. Mothers especially valued access to information and peer support programs, which contributed to family reorganization and improved caregiving strategies. These interventions, based on the exchange of experiences, have proven effective in reducing emotional distress and strengthening family resilience^(12,21,28,29)^. In this context, mental health nurse specialists can play a leadership role in the implementation and expansion of these programs.

Parental beliefs about their children’s abilities directly influence the organization of care and the incorporation of treatment into the routine. In the present study, this perception supported a management mindset focused on controlling the condition, even in the face of exhaustion. Studies indicate that this parental view is central to family adjustment to mental health demands^(5,12,18,30)^.

Emotional dysregulation in adolescents-expressed through self-harm, suicidal ideation, and aggression-has been described as one of the greatest challenges faced by families. Constant vigilance has generated chronic tension and harmed caregivers’ health and lives. Studies support these perceptions, associating such difficulties with increased burden and the emergence of a parental mindset marked by doubt about one’s own capacity for care^(2,3,19,31)^. Studies indicate that strengthening parental control can be achieved through interventions focused on developing skills, improving communication, and empowering caregivers. Furthermore, early treatment of emotional dysregulation tends to prevent risky behaviors and minimize future mental health complications^(26,28,29)^.

Family burden was intensified by the absence of public support and the difficulty of accessing qualified professionals. Families pointed to the lack of a structured network as an obstacle to the continuity and effectiveness of care. These findings are supported by studies that advocate for public policies that expand access to mental healthcare in adolescence, with positive impacts on public health and the care system^(32,33)^. Existing studies also indicate that professionals need to develop specific skills to recognize and manage such demands, as the resources and barriers faced by families directly influence how care is integrated into daily life^(22,26,31)^.

Family mutuality emerged as a central factor in daily coping. Couples with strong bonds before diagnosis reported that grief strengthened their caregiving partnership. In the absence of a father, stepfathers and grandparents took over. The literature reinforces that collaboration between partners favors more positive adaptive processes^(5,26,34)^, highlighting the increased difficulty faced in single-parent contexts, where there is less availability of support and resources, which reinforces the importance of interventions aimed at promoting family cohesion and belonging in the face of chronic care demands^(15-17,35)^.

Families reported beliefs and values that guide care, focusing on adolescents’ well-being, safety, and development. Their engagement in seeking specialized treatment was highlighted, revealing a resilient attitude. These findings converge with studies that recognize the family’s role as therapeutic support, a protective factor, or, in some cases, as a risk factor or trigger for symptoms^(25,30,36)^.

Despite support from other family members, maternal overload remains evident, with mothers playing the primary role in caregiving and coordination with services^(12,25,30)^. When present, fathers were considered fundamental for adherence to treatment and strengthening of bonds, which is corroborated by a study that associates their involvement with better psychosocial outcomes^(37)^.

Furthermore, the self-care strategies described by participants show that the impact of mental disorders on family dynamics tends to be more intense than in other chronic conditions, especially on parents, who report high levels of psychological distress, with a risk of emotional exhaustion in the absence of adequate support^(38)^. Reports from families revealed significant adjustments in domestic and professional routines, confirming findings that highlight the impact of the condition on family dynamics^(12,25,30)^.

The unpredictability of symptoms required a reorganization of tasks, improved communication, and family routine reconfiguration, often at the expense of caregivers’ needs. This data reinforces the importance of including family members in the therapeutic process, with emotional support and interventions sensitive to the specificities of each family unit^(39)^. Continuous care, especially without formal support, imposes constant decisions about time and resources, generating significant personal and professional sacrifices^(19,12,26)^.

Families reported that mental health disorders reshape routines and relationships, while caregiving is gradually incorporated into daily life. Some highlighted improvements in adolescents’ self-care and greater clinical stability, reinforcing gratitude for family dedication. Studies support the idea that small progress can boost the resumption of previously unfeasible activities and guide mental health interventions^(12,25,40)^.

In families with adolescents experiencing clinical instability, the focus on the condition intensifies. Caregivers report feeling like they are “walking on eggshells”, indicating hypervigilance in the face of the unpredictability of symptoms. Although associated with submissiveness in other contexts, this stance may reflect a refined relational skill, aimed at preventing crises and preserving bonds. Despite the high emotional cost, this management reveals the resilience and competence developed by families in the face of the complexity of care^(5,24)^.

Expectations for the future oscillate between adolescents’ desire for autonomy and parents’ fears about the persistence of dysfunctional symptoms and behaviors into adulthood. Family accounts converge with previous studies showing that mental health conditions can compromise independence, affecting decision-making, self-control, self-esteem, and, in some cases, intensifying impulsivity and risk-taking^(12,25,30,40)^. This scenario is experienced with anguish, especially in cases involving suicidal ideation and treatment interruption, as reported by Sofia’s family. The literature indicates that uncertainty about the future and the severity of symptoms fuel hopelessness, weakening the ability to project and maintain long-term plans^(19)^. In this context, it is up to nurses to maintain a sensitive clinical perspective, anchored in the advances already achieved, to restore hope and give new meaning to care as a continuous and achievable process.

In summary, understanding the experience of families caring for adolescents with mental disorders requires recognizing the complexity of daily management, parental emotions, and family reorganization. This study highlights not only the challenges but also the emerging strengths of bonding, learning, and resilience. By illuminating these experiences, it reinforces the need for sensitive clinical practices, inclusive public policies, and interventions that embrace family singularities, promoting shared, continuous, and humanized mental healthcare.

### Study limitations

Family experiences reveal unique dynamics, shaped by sociocultural, economic, and affective contexts that mold the care and perception of the adolescents’ condition, limiting the generalization of the findings. Furthermore, the absence of adolescents’ direct perspective points to the importance of broadening the viewpoint, incorporating their voices in understanding family management.

### Contributions to nursing, health or public policy

This study offers a sensitive overview of family experiences in the management of mental disorders in adolescents, with important implications for nursing practice and health. By recognizing these experiences as legitimate knowledge, professionals can integrate them into listening and care, promoting more comprehensive, responsive, and humanized care, attuned to families’ needs and the complexity of mental health.

## FINAL CONSIDERATIONS

Family members’ perspective, anchored in the FMSF, provided a deeper understanding of how mental health management is constructed and understood within the family context. Furthermore, it offered insights into possible personalized interventions that can be implemented within the healthcare network by the multidisciplinary team, especially nurses, throughout the care journey of these families. The findings highlight the urgent need for mental healthcare services, led by nurses, that assist families in adjusting their routines, promoting resilience and functionality through psychoeducation, problem-solving, and actions to reinforce strengths and mitigate problematic aspects of family management. These services are essential given the complex impact of mental health conditions on family dynamics, which requires adaptation, support, and understanding from both the family and society. It is recommended that future research broaden the understanding of family management by incorporating the perspectives of the adolescents themselves and the professionals involved in their care, as well as longitudinal studies that explore the evolution of these dynamics over time. Furthermore, multicenter investigations can contribute to comparing regional realities and identifying contextual factors that influence coping and family resilience.

## Data Availability

The research data are available in a repository: https://doi.org/10.25824/redu/NYXWBB.
