# Supplementary material for: Family experiences in managing mental disorders in adolescents
Source: Rev Bras Enferm. 2026 Jul 24;79:e20250312. doi: 10.1590/0034-7167-2025-0312 (PMC13403639; doi:10.1590/0034-7167-2025-0312)
Supplement: Translation

# Translation: Experiências das famílias no manejo do transtorno mental em adolescentes

**DOI:** 10.1590/0034-7167-2025-0312pt

**Authors:** Roberto Corrêa Leite, Maria Giovana Borges Saidel, Edirlei Machado dos Santos, Heloisa Garcia Claro Fernandes, Luciana de Lione Melo, Maria Cristina Mazzaia, Michelle Ferraz Martins Jamarim, Claudinei José Gomes Campos

**Affiliations:** I Universidade Estadual de Campinas. Campinas, São Paulo, Brasil; II Universidade Federal de Mato Grosso do Sul. Três Lagoas, Mato Grosso do Sul, Brasil; III Universidade Federal de São Paulo. São Paulo, São Paulo, Brasil

**Keywords:** Adolescente, Transtornos Mentais, Família, Relações Familiares, Saúde Mental.

## Abstract

**Objetivos:**

compreender a experiência de manejo familiar do transtorno mental em adolescentes à luz do *Family Management Style Framework*.

**Métodos:**

estudo de caso qualitativo, realizado com seis famílias entre julho e novembro de 2022. As entrevistas foram analisadas, segundo o modelo híbrido de análise temática, com apoio do NVivo^®^ 15.

**Resultados:**

as famílias compreendem o transtorno mental como uma condição marcada por estigma e desafios diários. A identidade do adolescente transita entre normalidade e vulnerabilidade, orientando os cuidados à segurança e ao bem-estar. As mudanças na rotina refletem o esforço para lidar com a imprevisibilidade, enquanto as expectativas futuras valorizam a autonomia e a saúde mental do adolescente.

**Considerações Finais:**

o estudo oferece subsídios para intervenções que promovam resiliência e funcionalidade familiar, com base na psicoeducação, resolutividade, reforço de pontos fortes e mitigação de aspectos problemáticos de manejo familiar.

## INTRODUÇÃO

Alterações biopsicossociais na adolescência podem comprometer o desenvolvimento cerebral, favorecendo o surgimento de transtornos mentais^(1)^. Nesse cenário, a regulação emocional é frequentemente afetada, dificultando a tolerância a estados emocionais intensos e persistentes^(2)^. A desregulação emocional se associa à ideação e tentativas de suicídio^(3)^, além de comportamentos como instabilidade de humor, explosões de raiva e agressividade^(2)^, representando um desafio para adolescentes e suas famílias.

Em 2021, cerca de um em cada seis jovens brasileiros entre 10 e 19 anos vivia com algum transtorno mental^(4)^, sendo essa a faixa etária mais vulnerável a autolesões, depressão e suicídio. Metade dos casos surge por volta dos 14 anos, mas permanece sem diagnóstico ou acompanhamento profissional. A depressão está entre as principais causas de incapacidade, e o suicídio é a terceira causa de morte entre jovens de 15 a 29 anos^(1)^.

Diante dessas condições, o estilo de manejo familiar influencia a adaptação individual e do sistema familiar^(5)^. No caso dos transtornos mentais, os pais enfrentam sintomas graves, como alucinações, delírios, isolamento e alterações de humor^(6)^. Estratégias mais eficazes incluem apoio emocional, tratamento adequado e interações empáticas, enquanto atitudes de controle voltadas à mudança de comportamento se mostram menos efetivas^(7)^. O manejo familiar diz respeito às ações e respostas da família diante da doença e do cuidado em saúde, sendo central no *Family Management Style Framework* (FMSF)^(8)^, que compreende três componentes: definição da situação; comportamentos de manejo; e consequências percebidas. Esse modelo possibilita compreender como as famílias lidam com as condições crônicas ao longo do ciclo vital^(5)^.

A definição da situação abrange significados subjetivos sobre o cuidado, incluindo a identidade do adolescente, visão da condição, mentalidade de manejo e mutualidade parental. Os comportamentos de manejo dizem respeito às condutas cotidianas, como filosofia parental e abordagem do cuidado. Já as consequências percebidas se referem aos impactos atuais e expectativas futuras da família^(5)^.

Embora o FMSF seja amplamente aplicado em condições crônicas^(8)^, sua utilização no contexto dos transtornos mentais na adolescência ainda é incipiente. Este estudo adota o FMSF para compreender como as famílias conduzem o cuidado cotidiano do adolescente com transtorno mental, destacando a necessidade de que os profissionais de saúde estejam preparados para apoiar o manejo dessas situações complexas.

## OBJETIVOS

Compreender a experiência de manejo familiar do transtorno mental em adolescentes à luz do FMSF.

## MÉTODOS

### Aspectos éticos

O estudo foi aprovado pelo Comitê de Ética em Pesquisa da Universidade Estadual de Campinas (UNICAMP), de acordo com o Certificado de Apresentação para Apreciação Ética n^o^ 47887321.6.0000.5404. As entrevistas foram realizadas apenas após a leitura e assinatura do Termo de Consentimento Livre e Esclarecido (TCLE) pelas famílias participantes.

### Tipo de estudo

Trata-se de pesquisa qualitativa^(9)^, que utilizou o FMSF^(8)^ como referencial teórico, conduzida por meio de estudo de caso do tipo múltiplos e holístico, apropriado para a investigação aprofundada de fenômenos em contextos de vida real^(10)^. As famílias constituíram o contexto dos casos, tendo o manejo familiar como unidade de análise. O estudo seguiu as orientações do *Consolidated Criteria for Reporting Qualitative Research* (COREQ), recomendadas para delineamento e análise em pesquisas qualitativas.

### Cenário do estudo

O estudo foi conduzido em ambiente virtual, no território nacional e domicílio da família participante. Foi escolhido um local em que participante e pesquisador se sentiam mais seguros, confortáveis e com garantia de privacidade.

### Fonte de dados

Foram incluídas famílias com pelo menos um adolescente entre 13 e 17 anos, com diagnóstico médico de transtorno mental confirmado por psiquiatra, em acompanhamento ambulatorial ou terapêutico há pelo menos seis meses. As famílias deveriam residir no território nacional, ter acesso à internet e condições para participar de entrevistas virtuais. Foram excluídas famílias cujos adolescentes apresentavam deficiência intelectual grave que inviabilizasse a comunicação ou aquelas que, após o primeiro contato, manifestaram desconforto ou desistência em participar do estudo. A coleta de dados foi realizada via plataforma *Zoom* ^®^, conforme diretrizes para pesquisas virtuais^(11)^, por meio de duas entrevistas semiestruturadas com cada família, assegurando credibilidade, confiabilidade e triangulação dos dados^(10)^. O roteiro seguiu as dimensões do FMSF: definição da situação; comportamentos de manejo; e consequências percebidas^(5)^. Participaram seis famílias com filhos adolescentes diagnosticados com transtornos mentais, residentes em diferentes regiões do Brasil e com acesso à internet. Os adolescentes foram identificados por nomes fictícios (Adam, Felipe, Sofia, Henrique, Aurora e Bela), e os pais, pelas iniciais e vínculo (ex.: “Mãe” ou “Pai”).

### Coleta e organização dos dados

A coleta de dados foi realizada pelo pesquisador principal, entre julho e novembro de 2022, em três etapas. A primeira etapa incluiu uma entrevista para a elaboração dos genogramas, ecomapas^(12)^ e da história de vida das famílias. Na segunda, aplicaram-se entrevistas semiestruturadas baseadas no FMSF, com duração média de 78 minutos. A terceira etapa consistiu em contato telefônico para esclarecimentos e encerramento dos casos. Foram conduzidas duas entrevistas, via *Zoom* ^®^, com cada uma das seis famílias, sendo 11 entrevistas individuais e uma entrevista com um casal, totalizando 12 entrevistas com sete familiares (seis mães e um pai). Os áudios foram transcritos e validados pelo *WhatsApp* ^®^, sem correções. A amostra foi intencional, com recrutamento por *snowball* ^(13)^ e encerrada quando as entrevistas alcançaram a saturação teórica^(9)^. A primeira família (família de Adam) foi indicada por uma enfermeira especialista em saúde mental e, assim como as demais, recebeu um convite para participar da pesquisa por meio de carta enviada pelo *WhatsApp* ^®^, após a confirmação dos critérios de inclusão. O convite continha as informações preliminares sobre a pesquisa, além do *link* do *Google Forms* ^®^ de acesso ao TCLE. Após o consentimento do TCLE, evidenciado pelo aceite e assinatura digital do participante, foi agendada a data da primeira entrevista. Para confirmar a saturação teórica, foram realizadas duas entrevistas adicionais (uma individual e uma com casal) após a quarta família. Como não surgiram novos temas ou contribuições relevantes às categorias analíticas a partir dessas entrevistas, constatou-se a saturação teórica, evidenciada pela repetição das narrativas e estabilidade das interpretações.

### Análise dos dados

Optou-se pelo modelo híbrido de análise temática, que combina codificação dedutiva e indutiva para identificar temas relevantes ao fenômeno estudado^(14)^. Os códigos dedutivos foram elaborados com base nas dimensões do FMSF^(5)^ e organizados em um *template* no NVivo^®^ por meio de matriz estrutural. A análise indutiva permitiu a inclusão do código “mutualidade na família”, respaldado pelas autoras do FMSF^(5,8)^ e por estudos com famílias em outras condições crônicas^(15-17)^. As transcrições foram exaustivamente codificadas, e os temas foram analisados e interpretados à luz do referencial. Também foi utilizada a nuvem de palavras no NVivo^®^ como recurso complementar de interpretação. A análise foi conduzida pelo pesquisador principal e validada por membros do grupo do Núcleo de Pesquisa e Estudos Qualitativos em Saúde (NUPEQS)/UNICAMP.

## RESULTADOS

Foram entrevistados sete familiares de seis adolescentes com transtornos mentais, residentes em São Paulo (três famílias), Paraíba, Rio de Janeiro e Pernambuco. Os participantes tinham entre 36 e 45 anos, sendo seis mães e um pai, pertencentes majoritariamente a famílias nucleares (três), além de duas recasadas e uma adotiva. Todos possuíam ensino superior completo e acesso a plano de saúde. Os adolescentes, entre 13 e 17 anos, incluíam um homem trans, dois meninos e três meninas, diagnosticados com transtorno do espectro autista, transtorno do déficit de atenção, hiperatividade transtorno afetivo bipolar, transtorno de ansiedade generalizada, depressão e transtorno de personalidade *borderline*. Em todas as famílias, as mães eram as principais cuidadoras dos adolescentes, mas nas famílias de Felipe e Bela, contava-se também com o apoio dos avós. A análise no NVivo^®^ destacou a palavra “condição” como a mais recorrente (0,23%) na nuvem de palavras (Figura 1), refletindo sua centralidade na experiência familiar e sua articulação com os demais sentidos evocados nos *verbatins*.

**Figura 1:** Nuvem das 20 palavras mais citadas, com um comprimento mínimo de oito caracteres, nas entrevistas das famílias participantes da pesquisa, Campinas, São Paulo, Brasil, 2025

A experiência de manejo familiar foi analisada com base nos componentes e dimensões do FMSF. Quanto à identidade do adolescente, algumas famílias adotaram uma visão mais otimista, associando a condição à normalidade possível. Outras enfatizaram limitações e impactos na socialização, nos aspectos psicossociais e nos comportamentos disfuncionais.

[...] *então, eu procuro, fora as necessidades particulares dos transtornos que são certos, nas condutas dele* [Felipe], *que a gente já sabe. Eu tento enxergá-lo de uma maneira normal e ver os desejos de socialização, as dificuldades que ele tem como as de outros adolescentes.* (Mãe, Família de Felipe)

*Aí, eu acho que a gente* [pais] *define que a Aurora teve sua adolescência roubada. Roubada totalmente, porque se foi* [...] *ela já não era de sair, aí a turma estava começando aquela fase de festa de 15 anos* [...]. *Aí nisso ela adoeceu; a turma* [amigos adolescentes] *continuaram a vida; ela, não!* [...] *mas é assim, ela perdeu essa fase.* (Mãe, Família de Aurora)

*Eu acho que ele* [Henrique] *não vivencia muito algumas coisas de adolescente. A cabeça dele funciona muito de outra forma.* [...] *uma vez ele me falou que ele tem que se esforçar para não pensar em se matar; isso, para mim, já é uma coisa assim muito triste de ouvir de um adolescente. Então, eu acho que as preocupações dele são muito grandes para um menino de 16 anos.* (Mãe, Família de Henrique)

A visão das famílias sobre a condição é impactante e atravessada por sentimentos intensos relacionados à causa, gravidade e prognóstico do transtorno mental. A trajetória envolve o luto pelo filho idealizado, a culpa, o estigma e a busca por conhecimento para compreender e manejar melhor a situação.

*Eu descobri o diagnóstico com 2 anos. Horrível, horrível, horrível! Eu fiquei muito mal* [...] *eu ia trabalhar no automático, sofri muito* [...] *então, assim, a parte emocional, eu acho que eu vivi um luto.* (Mãe, Família de Adam)

*Às vezes, eu fico assim, Roberto* [pesquisador]. *Eu fico dizendo: “Onde foi que eu errei? O que eu errei? Onde foi? Qual momento?”. Tentando identificar cronologicamente* [...]. (Pai, Família de Bela)

*Ele* [transtorno mental] *não é visto. Ele não é falado, não vou generalizar, mas para um número grande de pessoas, ele é um problema que parece bobeira. Deixa para lá, levanta sacode a poeira e vamos lá* [...] *é isso. É no trabalho de quem tem burnout ou que tem uma depressão, ou na escola, como ela* [Aurora] *vivenciava*. (Mãe, Família de Aurora)

[...] *eu comecei a pesquisar sobre o assunto, e comecei a compreender mais um pouco a doença, as necessidades. Então, a psicoeducação é muito necessária,* [...] *não existe uma psicoeducação dos pais.* [...] *inclusive, foi um ponto para mim muito importante quando eu entrei no Family Connections* [treino de habilidades para cuidadores de pessoas com condições de saúde mental] *como familiar. Eu fiquei: “Nossa, tem tanta gente passando por tanta coisa parecida com a gente, não é”. São as mesmas dúvidas que a gente pensa* [...]. (Mãe, Família de Felipe)

A mentalidade de manejo revela como os pais percebem suas habilidades, desafios e apoios no cuidado. Apesar da exaustão e das incertezas, reconhecem o manejo como um processo contínuo, que exige paciência e perseverança para garantir a segurança dos filhos.

*Muito difícil, mas muito difícil mesmo* [cuidar de Sofia]*! Eu achava que eu não teria essa força aí, esse discernimento para poder fazer as coisas que eu tenho que fazer hoje. É um cuidar que você tem que pensar em tudo, em tudo que você vai fazer, desde um simples banho, que lá dentro ela pode se cortar, até o dormir, que ela pode acordar e, então, é muito, muito difícil! Tudo o que a gente propõe é um “não”! Então, a gente tem que ir com muita calma, às vezes não ir, às vezes não fazer, e assim vivendo.* (Mãe, Família de Sofia)

A dificuldade em controlar as crises de desregulação emocional e prever seus gatilhos gera medo, frustração e ansiedade, mantendo os pais em estado de hipervigilância. As famílias também relatam a falta de suporte do poder público e de profissionais de saúde diante do sofrimento psíquico e do impacto psicossocial do cuidado.

*E eu pedia para ela* [Bela], *pelo amor de Deus e aos prantos soluçando, para não fazer mais isso* [autolesão], *para não tentar* […] *só de falar, eu começo a marejar os olhos! É isso, às vezes fica difícil de você identificar o gatilho, que pode ou o que não pode causar aquele, vamos dizer, aquele é o estopim, não é?* (Pai, Família de Bela)

*Extremamente exaustivo* [cuidar de Felipe]*. Tem um impacto na sua vida pessoal, profissional e na sua saúde física e mental, um impacto profundo, e nós fazemos isso com cada dia menos ajuda do poder público. A saúde mental ainda é vista como um problema seu, e zero apoio por aqui* [cidade onde ela mora], *que vai do SAMU ao lugar que você leva.* [...] *onde é que a gente está, não é? O povo* [profissionais de saúde]*, que era para ser especializado em atender uma coisa dessa, que dificuldade!* (Mãe, Família de Felipe)

De acordo com a mutualidade parental, os pais se tornaram mais unidos na experiência, resultando em maior conexão, comunicação e reciprocidade entre o casal.

[...] *a gente sempre foi um casal bem próximo, mas eu acho que agora a dor* [da condição de Aurora], *não é? A dor faz com que você esteja junto e aguente, e dando apoio um para o outro.* (Mãe, Família de Aurora)

*Mais união! Eu acho que o relacionamento já era um relacionamento bem sólido, e quando você tem um filho, seja qualquer condição que ele tem, se ele não for um relacionamento sólido, não há essa doação e essa divisão* [...] *e eu tenho muito apoio do meu marido, graças a Deus!* (Mãe, Família de Adam)

Em outras famílias, o manejo da condição foi centralizado na família extensa ou no padrasto, devido à ausência do pai biológico, o que caracterizou a mutualidade familiar.

*Mas hoje a gente* [família extensa] *também tem união e é bastante fortalecida nesse aspecto também.* [...] *e hoje somos, acho que também, por tudo que a gente já passou, somos ainda mais unidos.* (Mãe, Família de Felipe)

*Acaba que é muito nós dois* [ela e o padrasto] [...] *a gente conversa muito sobre tudo. Eu procuro deixar ele sempre a par do que eu fico sabendo, do que acontece* [...]. (Mãe, Família de Henrique)

Os comportamentos de manejo refletem a filosofia parental e a abordagem adotada. Todas as famílias demonstraram forte crença na eficácia das intervenções multiprofissionais e compromisso com o desenvolvimento emocional, cognitivo e comportamental dos filhos.

*Mas a gente a levou em um outro médico especialista, que foi aí que ele começou a trabalhar com a hipótese de borderline. Trocou as medicações, mas mesmo assim não deu certo, e a gente precisou interna-la na primeira clínica.* (Mãe, Família de Sofia)

[...] *é uma fonoaudióloga, um psicólogo individual comportamental, e aí eu fui cavando os meus direitos procurando bem o que era de melhor.* (Mãe, Família de Adam)

As mães vivem em constante estado de prontidão, sempre à espera da próxima crise, e enfrentam a imprevisibilidade no cotidiano. Essas experiências resultam em desgaste emocional e impactam negativamente a qualidade de vida. Com isso, elas focam em estratégias de autocuidado para fortalecer sua saúde mental, diante da sobrecarga física e emocional que enfrentam ao cuidar dos adolescentes.

*Eu costumo pensar que a gente está sempre em alerta, sempre esperando a próxima crise, esperando a próxima situação, esperando o que vai acontecer, como vai ser. Agora eu estou conseguindo lhe dar um pouco melhor com isso, com a terapia, mas, há um tempinho atrás, eu sempre tinha na minha cabeça que a qualquer momento alguém ia me ligar e dizer que meu filho fez uma besteira* [tentativa de suicídio]. (Mãe, Família de Henrique)

*Porque eu não via luz no fim do túnel mais. Eu já estava numa questão de desespero! Inclusive, eu tive muitos pensamentos suicidas. Não é aquele pensamento que você parte para a prática que você planeja* [...] *mas aquela coisa que você deseja. Eu preferiria estar morta por ter passado por tudo isso. Entendeu?* [...] *então, eu fui em busca de ajuda, porque, senão, eu não ia conseguir ajudá-lo* [Felipe]. (Mãe, Família de Felipe)

A abordagem de manejo mostrou que as mães ajustaram suas rotinas de trabalho para acompanhar os adolescentes nas consultas e terapias, garantindo apoio contínuo e prevenção de comportamentos de risco. As crises de desregulação também demandaram mudanças na dinâmica familiar, com ênfase na observação de limites e comunicação empática.

*Eu já desisti de empregos. Já optei por outros, porque o F.* [pai]*, como ele tem um cargo administrativo, é sempre esse horário, das 08:00 às 17:00, mas eu já troquei de emprego* [...] *já tive emprego de seis horas, que era das 14:00 às 20:00, para dar suporte no horário da manhã. Enfim, foi nesse sentido. Já abri mão, já fiquei em casa, já pedi a conta* [demissão]. (Mãe, Família de Adam)

[...] *então, eu já tinha que estar atrás de médico e atrás de psicólogo. Era problema com ele* [Felipe], *problema na escola, e aquilo me demandava tanto tempo, que eu não tinha condição de conciliar aquilo com a minha vida profissional. Quando eu tentei* [trabalhar], *eu vi uma piora tão grande no menino que eu tive que parar.* (Mãe, Família de Felipe)

*Aquele puxão de orelha, e aí você diz: “Não vamos entrar como um elefante numa loja de cristal, a gente tem que ir mais devagarzinho, para não ser o gatilho”* [desregulação emocional]. *Eu acho que é ter essa sensibilidade de pegar meio leve* […]. (Pai, Família de Bela)

As consequências percebidas envolvem o foco das famílias no cuidado dos adolescentes em meio à imprevisibilidade e às interferências cotidianas. A rotina exige ações cautelosas, frequentemente descritas como “pisar em ovos”. Ainda assim, algumas mães relatam ajustes progressivos ao longo do tempo, com percepção positiva das mudanças.

*Enfim, não dá para você ter uma vida muito estabilizada e emocionalmente estruturada por muito tempo. Parece que todo momento alguma coisa vai mudar e você tem que ir mudando os planos* [...]. (Mãe, Família de Adam)

[...] *tudo, tudo, tudo, tudo que você imaginar, mudou. Eu acho que não existe nada igual como antes* [...] *mesmo ela* [Sofia] *estando internada, hoje a gente ainda continua com a mesma rotina de cuidado* [...] *com medo do celular tocar e a gente precisar sair correndo. É aquilo que a gente sempre ouve. Parece que a gente está vivendo para ficar pisando em ovos o tempo todo*. (Mãe, Família de Sofia)

*Totalmente, interfere de tudo* [cuidados com Felipe] [...] *então, é uma rotina completamente diferente.* [...] *hoje, quando eu chego em casa,* [...] *eu vi que ele tinha estendido a roupa dele, que ele tinha tomado o remédio dele, sem eu mandar nada, porque eu estava dormindo. Então, sou muito agradecida por tudo, porque eu sei o que a gente* [família] *já passou* [...]. (Mãe, Família de Felipe)

A análise das expectativas futuras evidencia o desejo de que os adolescentes alcancem autonomia e independência, ao mesmo tempo em que expressa o medo e a preocupação dos pais em relação à possibilidade de interrupção do tratamento e ao consequente aumento do risco de suicídio.

*A minha expectativa em relação ao Adam é que ele seja funcional! Que ele consiga em atividades básicas viver, no ir e vir, de uma maneira autônoma. Eu acho que é isso! Em relação à minha família, é acompanhar esse processo de evolução dele, é ter a satisfação de, no futuro, ver que nada, que nada, foi em vão. Entendeu? Que deu tudo certo!* (Mãe, Família de Adam)

[...] *eu quero que ela* [Aurora] *não dependa, que ela seja independente, ela vai e faça as coisas. Pode vir para a minha casa, não tem problema, estar aqui, mas tem a sua vida, tenha seus filhos. Pode trazer eles para eu ver, não quero opinar também* [...]. (Mãe, Família de Aurora)

*Mas você tem alguma expectativa em relação ao futuro da Sofia?* (Pesquisador)

*Não, eu só quero que ela fique viva! Que ela fique viva! Porque eu tenho certeza que a partir do momento que ela parar de tomar o lítio* [medicação], *ela volta com a ideação suicida.* (Mãe, Família de Sofia)

## DISCUSSÃO

A análise dedutiva e indutiva, orientada pelo FMSF, revelou que a identidade do adolescente oscila entre percepções de normalidade e limitações impostas pelo transtorno mental. A normalização surgiu como estratégia de enfrentamento e adaptação ao cotidiano. Em contrapartida, o medo constante de crises e comportamentos de risco à vida comprometeram a funcionalidade familiar, intensificando a vigilância. Tais achados dialogam com a literatura, que reconhece a normalização como recurso de enfrentamento^(18)^, enquanto a exposição contínua ao sofrimento tende a fragilizar os recursos adaptativos das famílias^(19,20)^.

A visão da condição mostrou o diagnóstico como uma ruptura dolorosa associada ao luto^(21)^ e à busca por conhecimento^(22)^. A negação apareceu como resposta inicial à perda do filho idealizado, cedendo espaço, com o tempo, à aceitação. Esses resultados convergem com estudos que entendem a negação como mecanismo de defesa e fase transitória do luto parental^(12,21,23,24)^. Esses estudos destacam a importância de os profissionais, especialmente enfermeiros, reconhecerem esse movimento como parte do processo adaptativo, oferecendo suporte com escuta validante e presença sensível.

A culpa foi recorrente nos relatos, refletindo uma responsabilização parental ligada ao desconhecimento sobre a condição. Crenças de que o desenvolvimento dos filhos depende apenas do amor e esforço dos pais intensificaram esse sentimento, levando à internalização do sofrimento^(19,25,26)^. Os enfermeiros desempenham um papel fundamental ao acolher as famílias com escuta qualificada e informações claras, para o alívio da culpa e ressignificação da experiência parental.

Além disso, os participantes relataram se sentir desamparados diante da invisibilidade e deslegitimação social das condições de saúde mental. Tais percepções foram associadas à vivência do estigma e à sensação de não pertencimento, que podem ser agravadas por um modelo biomédico que tende a categorizar os indivíduos como diferentes, contribuindo para o enfraquecimento dos vínculos sociais, da resiliência familiar e do processo de recuperação dos adolescentes^(21,27)^.

A falta de orientação profissional fez com que as famílias buscassem ativamente fontes de conhecimento. As mães valorizaram, especialmente, o acesso a informações e a programas de suporte de pares, que contribuíram para a reorganização familiar e aprimoramento das estratégias de cuidado. Essas intervenções, baseadas na troca de experiências, demonstram eficácia na redução do sofrimento emocional e fortalecimento da resiliência familiar^(12,21,28,29)^. Nesse contexto, o enfermeiro especialista em saúde mental pode exercer papel de liderança na implementação e ampliação desses programas.

As crenças parentais sobre as capacidades dos filhos influenciam diretamente a organização dos cuidados e a incorporação do tratamento à rotina. No presente estudo, essa percepção sustentou uma mentalidade de manejo focada no controle da condição, mesmo diante da exaustão. Estudos apontam que tal visão dos pais é central para o ajustamento familiar às demandas da saúde mental^(5,12,18,30)^.

A desregulação emocional dos adolescentes - expressa por autolesão, ideação suicida e agressividade - foi descrita como um dos maiores desafios enfrentados pelas famílias. A vigilância constante gerou tensão crônica e prejuízos à saúde e à vida dos cuidadores. Estudos corroboram essas percepções, associando tais dificuldades ao aumento da sobrecarga e à emergência de uma mentalidade parental marcada pela dúvida sobre a própria capacidade de cuidado^(2,3,19,31)^. Estudos indicam que o fortalecimento do controle parental pode ser alcançado por meio de intervenções voltadas ao desenvolvimento de habilidades, melhoria da comunicação e empoderamento dos cuidadores, além de que o tratamento precoce da desregulação emocional tende a prevenir comportamentos de risco e minimizar complicações futuras em saúde mental^(26,28,29)^.

A sobrecarga familiar foi intensificada pela ausência de apoio público e pela dificuldade de acesso a profissionais qualificados. As famílias apontaram a falta de uma rede estruturada como entrave à continuidade e efetividade do cuidado. Tais achados são sustentados por estudos que defendem políticas públicas que ampliem o acesso à saúde mental na adolescência, com impactos positivos na saúde coletiva e no sistema de cuidado^(32,33)^. Estudos existentes também apontam que os profissionais precisam desenvolver competências específicas para reconhecer e manejar tais demandas, pois as facilidades e barreiras enfrentadas pelas famílias influenciam diretamente a forma como o cuidado é integrado ao cotidiano^(22,26,31)^.

A mutualidade familiar surgiu como fator central no enfrentamento cotidiano. Casais com vínculos sólidos antes do diagnóstico relataram que a dor fortaleceu a parceria no cuidado. Na ausência paterna, padrastos e avós assumiram esse papel. A literatura reforça que a colaboração entre parceiros favorece processos adaptativos mais positivos^(5,26,34)^, destacando a dificuldade acrescida enfrentada em contextos monoparentais, nos quais há menor disponibilidade de apoio e recursos, o que reforça a importância de intervenções voltadas à promoção da coesão e do pertencimento familiar diante das exigências do cuidado crônico^(15-17,35)^.

As famílias relataram crenças e valores que orientam o cuidado, com foco no bem-estar, segurança e desenvolvimento dos adolescentes. Destacou-se o engajamento na busca por tratamento especializado, revelando uma postura resiliente. Esses achados convergem com estudos que reconhecem o papel da família como suporte terapêutico, fator de proteção ou, em alguns casos, como elemento de risco ou desencadeador dos sintomas^(25,30,36)^.

Apesar do apoio de outros familiares, a sobrecarga materna permanece evidente, com as mães assumindo o papel principal no cuidado e na articulação com os serviços^(12,25,30)^. Quando presentes, os pais foram considerados fundamentais para a adesão ao tratamento e fortalecimento dos vínculos, o que é corroborado por estudo que associa seu envolvimento a melhores desfechos psicossociais^(37)^.

Além disso, as estratégias de autocuidado descritas pelos participantes evidenciam que o impacto dos transtornos mentais na dinâmica familiar tende a ser mais intenso do que em outras condições crônicas, especialmente sobre os pais, que relatam altos níveis de sofrimento psíquico, com risco de exaustão emocional diante da ausência de apoio adequado^(38)^. Os relatos das famílias revelaram ajustes significativos nas rotinas domésticas e profissionais, o que confirma achados que evidenciam o impacto da condição na dinâmica familiar^(12,25,30)^.

A imprevisibilidade dos sintomas exigiu reorganização das tarefas, aprimoramento da comunicação e reconfiguração da rotina familiar, frequentemente em detrimento das necessidades dos cuidadores. Esses dados reforçam a importância de incluir os familiares no processo terapêutico, com suporte emocional e intervenções sensíveis às especificidades de cada núcleo familiar^(39)^. O cuidado contínuo, especialmente sem apoio formal, impõe decisões constantes sobre tempo e recursos, gerando sacrifícios pessoais e profissionais relevantes^(19,12,26)^.

As famílias relataram que o transtorno mental reconfigura rotinas e relações, ao passo que os cuidados são gradualmente incorporados ao cotidiano. Alguns destacaram avanços no autocuidado dos adolescentes e maior estabilidade clínica, reforçando a gratidão pela dedicação familiar. Estudos sustentam que pequenos progressos podem impulsionar a retomada de atividades antes inviáveis e orientar intervenções em saúde mental^(12,25,40)^.

Nas famílias com adolescentes em instabilidade clínica, o foco na condição se intensifica. Os cuidadores relataram a sensação de “pisar em ovos”, indicando hipervigilância diante da imprevisibilidade dos sintomas. Embora associada à submissão em outros contextos, essa postura pode refletir uma habilidade relacional refinada, voltada à prevenção de crises e preservação dos vínculos. Apesar do alto custo emocional, esse manejo revela a resiliência e a competência desenvolvidas pelas famílias diante da complexidade do cuidado^(5,24)^.

As expectativas em relação ao futuro oscilam entre o desejo dos adolescentes por autonomia e o receio dos pais quanto à persistência dos sintomas e comportamentos disfuncionais na vida adulta. Os relatos familiares convergem com estudos prévios de que condições de saúde mental podem comprometer a independência, afetando a tomada de decisão, o autocontrole, a autoestima e, em alguns casos, intensificando a impulsividade e a exposição a riscos^(12,25,30,40)^. Esse cenário é vivido com angústia, sobretudo nos casos com ideação suicida e interrupção do tratamento, como descrito pela família de Sofia. A literatura aponta que a incerteza diante do futuro e a gravidade dos sintomas alimentam a desesperança, enfraquecendo a capacidade de projetar e manter planos de longo prazo^(19)^. Nesse contexto, cabe ao enfermeiro manter um olhar clínico sensível, ancorado nos avanços já conquistados, para resgatar a esperança e ressignificar o cuidado como um processo contínuo e possível.

Em síntese, compreender a experiência de famílias que cuidam de adolescentes com transtorno mental exige reconhecer a complexidade do manejo cotidiano, das emoções parentais e da reorganização familiar. Este estudo evidencia não apenas os desafios, mas também as potências emergentes do vínculo, da aprendizagem e da resiliência. Ao iluminar essas vivências, reforça-se a necessidade de práticas clínicas sensíveis, políticas públicas inclusivas e intervenções que acolham as singularidades familiares, promovendo um cuidado em saúde mental compartilhado, contínuo e humanizado.

### Limitações do estudo

As vivências das famílias revelam dinâmicas singulares, atravessadas por contextos socioculturais, econômicos e afetivos que moldam o cuidado e a percepção da condição dos adolescentes, limitando a generalização dos achados. Além disso, a ausência da perspectiva direta dos adolescentes aponta para a importância de ampliar o olhar, incorporando suas vozes na compreensão do manejo familiar.

### Contribuições para a área da enfermagem, saúde ou políticas públicas

Este estudo oferece um panorama sensível das vivências familiares no manejo do transtorno mental em adolescentes, com importantes implicações para a prática da enfermagem e saúde. Ao reconhecerem essas experiências como saberes legítimos, os profissionais podem integrá-las à escuta e ao cuidado, favorecendo uma atenção mais integral, responsiva e humanizada, sintonizada com as necessidades das famílias e a complexidade da saúde mental.

## CONSIDERAÇÕES FINAIS

A perspectiva dos familiares, ancorada no FMSF, proporcionou um entendimento aprofundado sobre como o manejo do transtorno mental é construído e compreendido no contexto familiar. Além disso, ofereceu subsídios para possíveis intervenções personalizadas que podem ser articuladas nas Redes de Atenção à Saúde pela equipe multidisciplinar, especialmente pelo enfermeiro, durante a jornada de cuidado dessas famílias. Os achados destacam a urgência de serviços de saúde mental, liderados por enfermeiros, que auxiliem as famílias na acomodação de suas rotinas, promovendo resiliência e funcionalidade por meio de psicoeducação, resolutividade e ações para reforçar os pontos fortes e mitigar aspectos problemáticos de manejo familiar. Esses serviços são essenciais diante da complexidade do impacto das condições de saúde mental na dinâmica familiar, que requer adaptação, suporte e compreensão tanto da família quanto da sociedade. Recomenda-se que futuras pesquisas ampliem a compreensão do manejo familiar incorporando as perspectivas dos próprios adolescentes e dos profissionais envolvidos no cuidado, bem como estudos longitudinais que explorem a evolução dessas dinâmicas ao longo do tempo. Além disso, investigações multicêntricas podem contribuir para comparar realidades regionais e identificar fatores contextuais que influenciam o enfrentamento e a resiliência das famílias.

**FOMENTO:** A pesquisa recebeu apoio da Coordenação de Aperfeiçoamento de Pessoal de Nível Superior.

## Data Availability

Os dados de pesquisa estão disponíveis em repositório: https://doi.org/10.25824/redu/NYXWBB.
